# Proteomics-based identification of VDAC1 as a tumor promoter in cervical carcinoma

**DOI:** 10.18632/oncotarget.10562

**Published:** 2016-07-13

**Authors:** Changlin Zhang, Wencheng Ding, Yuan Liu, Zheng Hu, Da Zhu, Xiaoli Wang, Lan Yu, Liming Wang, Hui Shen, Weican Zhang, Ci Ren, Kezhen Li, Danhui Weng, Wuguo Deng, Ding Ma, Hui Wang

**Affiliations:** ^1^ Department of Obstetrics and Gynecology, Tongji Hospital, Tongji Medical College, Huazhong University of Science and Technology, Wuhan, Hubei 430030, China; ^2^ Sun Yat-Sen University Cancer Center, State Key Laboratory of Oncology in South China, Collaborative Innovation Center of Cancer Medicine, Guangzhou, Guangdong 510060, China; ^3^ The State Key Laboratory of Biotherapy, West China Hospital, Sichuan University, Sichuan 610041, China; ^4^ Department of Obstetrics and Gynecology, Hebei Medical University, Shijiazhuang, Hebei 050017, China

**Keywords:** proteomics, immunohistochemistry (IHC), cervical cancer, VDAC1, HPV16 E7

## Abstract

We used oxidative isotope-coded affinity tags (OxICAT) to investigate the global redox status of proteins in human papillomavirus (HPV)-related cervical cancer cells, in order to identify a potential target for gene therapy. Voltage-dependent anion channel 1 (VDAC1) was found to be highly oxidized in HPV-positive cervical cancer cells. VDAC1 expression correlated significantly with the invasion of cervical cancer, the grade of cervical intraepithelial neoplasia (CIN) and the expression of HPV16 E7 in CIN. Knockdown of VDAC1 in cell lines increased the rate of apoptosis, while overexpression of the VDAC1 (respectively) partly reversed the effect. Thus, VDAC1 may promote the malignant progression of HPV-related disease, and treatments designed to suppress VDAC1 could prevent the progression of HPV-induced cervical disease.

## INTRODUCTION

Cervical cancer is the second most common malignant tumor after breast cancer in women worldwide [1]. Due to widespread practice of cervical screening, most cervical cancerous and precancerous lesions can be detected and treated early [2], which can significantly reduce cervical cancer incidence and mortality, However, the incidence in women younger than 35 years has been increasing [3]. At present, infection with HPV, especially persistent infection with high-risk HPV types 16 and 18, is the major cause of cervical precancerous lesions and cancer [1, 3]. HPV is a highly host-specific, non-enveloped, double-stranded circular DNA virus, and highly expresses E6 and E7, the primary oncogenes whose products are responsible for the initiation and progression of cervical cancer [4, 5].

Mitochondria not only provide energy for the biological activities of eukaryotic cells [6], but also perform important functions in cell differentiation, signal transduction and apoptosis [7], and are able to regulate cell growth and the cell cycle [8]. VDAC proteins, located in the outer membrane of mitochondria, constitute the mitochondrial permeability transition pore [9, 10]. Through the regulation of VDAC proteins and endometrial adenine nucleotide translocator, mitochondrial permeability transition pores open and close in a timely fashion to maintain the ion balance and membrane potential of the mitochondria [8, 11, 12]. VDAC has three isoforms: VDAC1, VDAC2 and VDAC3, all of which are important mitochondrial regulatory proteins that maintaining the normal microenvironment of the cell [9].

Our previous work has focused on the redox state changes of proteins in the course of virus-induced tumorigenesis [13–18]. In this study, we were curious to know which kinds of proteins undergo changes in redox state during cervical cancer caused by HPV infection. We used OxICAT technology to screen the candidate proteins involved in HPV infection and screened out VDAC1, VDAC2 and VDAC3. Reactive oxygen species can change the redox states of VDAC proteins and thus make cells more sensitive to apoptosis and autophagy [19–21]. HPV16 E7 is an oncoprotein that facilitates the development of cancer in cervical tissues infected with HPV16. Thus, we decided to explore the involvement of HPV16 E7 and VDAC proteins in the initiation and progression of cervical cancer. We also verified the effects of the selected proteins by IHC analysis in clinical cervical cancer tissues and other in vitro experiments. We found that VDAC1, one major protein of the VDAC protein family, may act as a tumor promoter in cervical cancer, and thus may be a novel target for the prevention and treatment of cervical carcinoma.

## RESULTS

### The OxICAT method screened out VDAC1, VDAC2 and VDAC3

**Figure 1:**
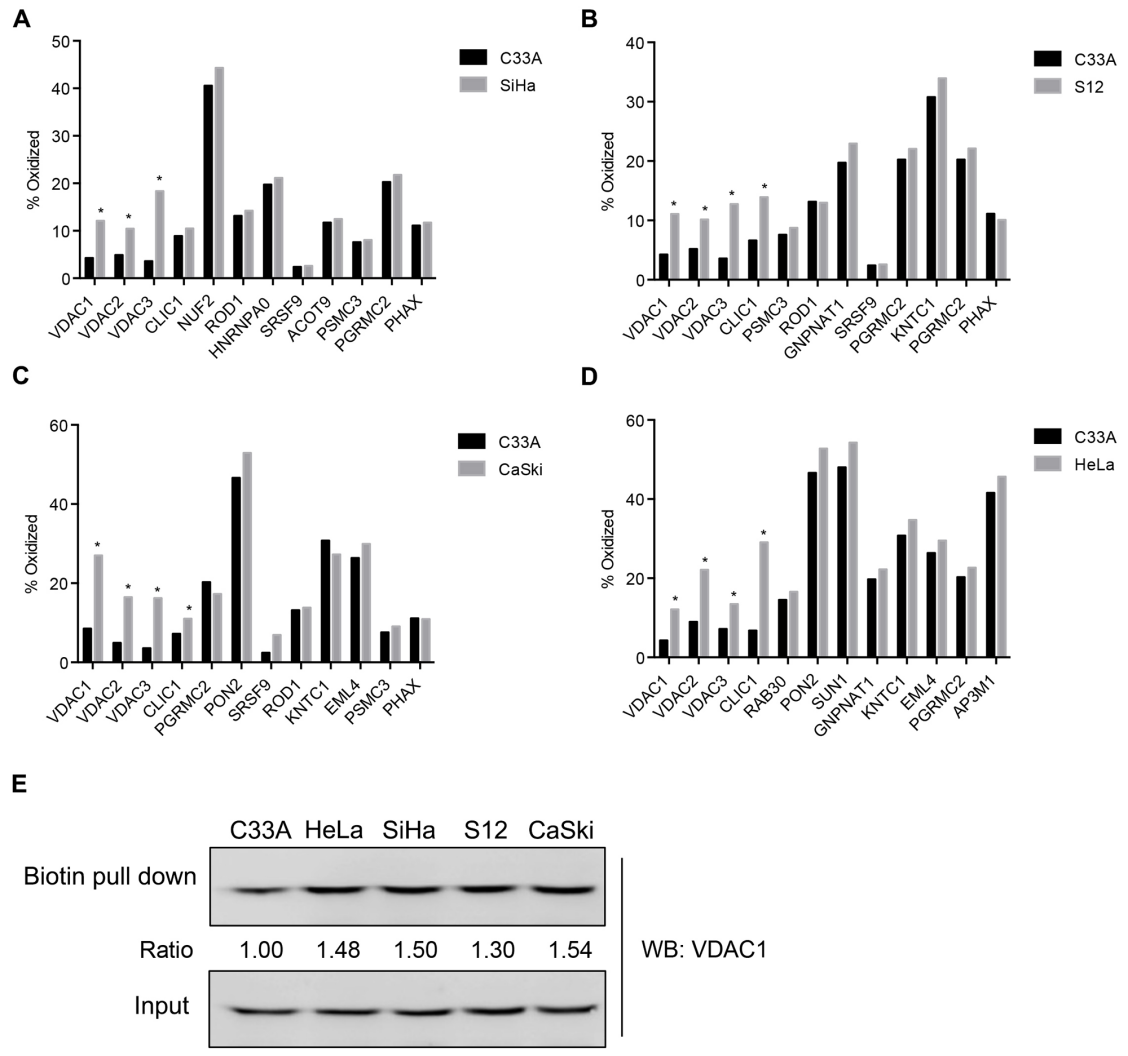
OxICAT screened out VDAC1, VDAC2 and VDAC3 in HPV-related cervical cancer cells Five cervical cancer cell lines, SiHa (HPV16-positive), S12 (HPV16-positive), CaSki (HPV16/18-positive), HeLa (HPV18-positive) and C33A (HPV-negative) were used to estimate the global status of proteins by OxICAT. **A.** The oxidation states of proteins in SiHa and C33A cell lines. **B.** The oxidation states of proteins in S12 and C33A cell lines. **C.** The oxidation states of proteins in CaSki and C33A cell lines. **D.** The oxidation states of proteins in HeLa and C33A cell lines. **E.** Validation of VDAC1 proteomic results by BIAM assays. All data are presented as the mean ± SD of three independent experiments, *P < 0.05.

In order to assess the redox states of proteins during the process of HPV infection in cervical cancer, we used OxICAT to quantitatively detect the global oxidation status of proteins in HPV-related cervical cancer cells. VDAC1, VDAC2 and VDAC3, the three isoforms of VDAC, were significantly oxidized in both HPV16-positive (SiHa, S12 and CaSki) and HPV18-positive (HeLa) cervical cancer cells compared with HPV-negative cervical cancer cells (C33A) (Figure 1A-1D). Except for CLIC1, we did not find significant differences in oxidation in other detectable cell energy- and cell survival-associated proteins (RAB30, PON2, NUF2, ROD1, and so on) between the HPV-positive and HPV-negative cervical cancer cell lines. To further validate the proteomics results for VDAC1, we performed the N-(biotinoyl)-N’-(iodoacetyl)ethylenediamine (BIAM) assay and found that VDAC1 was significantly oxidized in HPV-positive cells compared with C33A (Figure 1E). These data suggested that VDAC1 is significantly oxidized in both HPV16- and HPV18-positive cells.

### VDAC1 expression correlated with the invasion of cervical cancer

**Figure 2:**
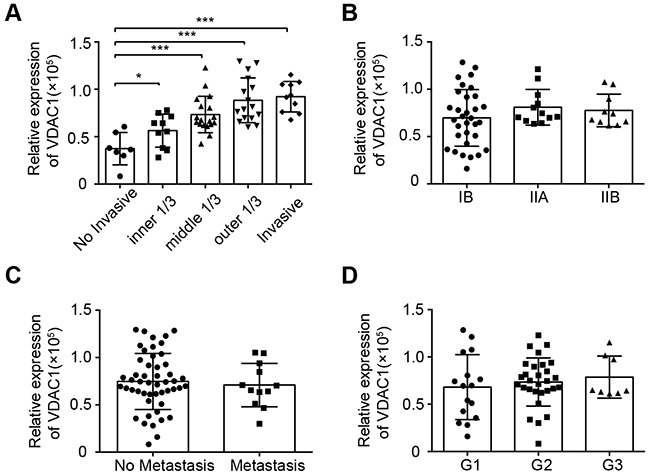
VDAC1 expression correlated with the invasive depths of cervical cancer tissues The expression of VDAC1 in cervical cancer specimens was detected by IHC staining, and the relationships between the levels of VDAC1 and clinicopathologic features were estimated. **A.** The relative expression of VDAC1 in cervical cancer tissues with different invasive depths. *, P < 0.05, ***, P < 0.001. **B.** The relative expression of VDAC1 in cervical cancer tissues according to FIGO staging. **C.** The relative expression of VDAC1 in cervical cancer tissues with or without metastasis. **D.** The relative expression of VDAC1 in cervical cancer tissues according to differentiation status: G1, well-differentiated; G2, moderately differentiated; G3, poorly differentiated. The data are presented as the mean ± SD with scatter plots representing the individual detailed protein levels.

HPV16 is the most common subtype present in biopsies from women with cervical squamous cell carcinoma. To detect the expression of the candidate proteins in HPV16-positive cervical cancer tissues, we used IHC staining to assess the expression of VDAC1, VDAC2, VDAC3 and HPV16 E7. Furthermore, we evaluated the relationships between the expression levels and clinicopathological parameters. The expression of VDAC1 correlated significantly with cervical cancer invasion (Figure 2A), but was not associated with staging, lymph node metastasis or the differentiation degree of cervical cancer tissue (Figure 2B-2D). VDAC2 and VDAC3 levels correlated significantly with metastasis, while neither were related to invasive depth, staging or differentiation degree (Supplementary Figure 1-2). These data suggested that the expression of VDAC1 may correlate with the invasion of cervical cancer. The clinicopathological parameters of the cervical tissue used in this assay are listed in Table 1 and Supplementary Table S1–S2.

**Table 1:** Basal characteristics of tissues used in this article

Characteristic		Number
Normal tissure		10
CIN		80
	CINI	47
	CINII	15
	CINIII	18
Cervical Cancer		57
Invasion depth		
	No invasive	6
	Inner1/3	9
	Middle1/3	18
	Outer1/3	14
	Invasive	10
FIGO Staging		
	IB	34
	IIA	13
	IIB	10
Metastasis		
	POS	11
	NEG	46
Pathological grading		
	G1	4
	G2	35
	G3	18

### HPV16 E7 expression was not related to clinicopathological parameters or VDAC1 expression in cervical cancer

**Figure 3:**
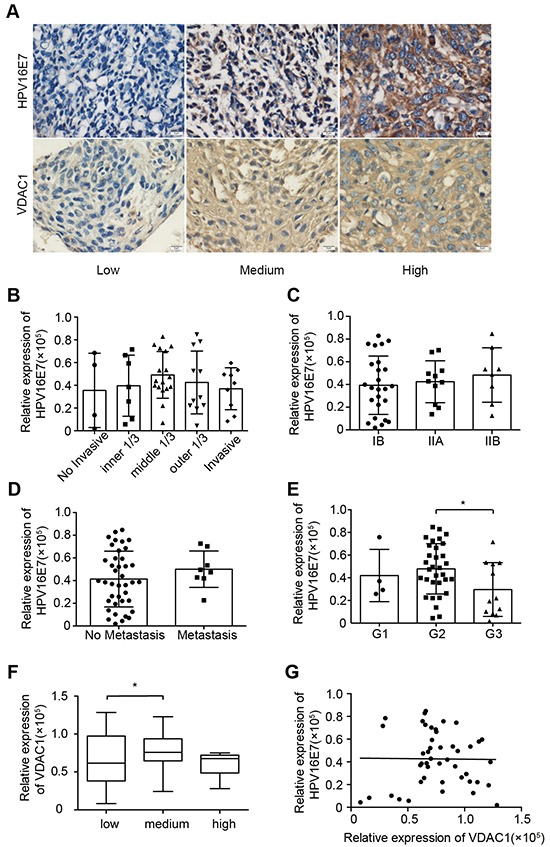
HPV16 E7 expression was not related to clinicopathologic features or VDAC1 expression in cervical cancer The expression of HPV16 E7 in cervical cancer specimens was detected by IHC staining, and the relationships between the expression of HPV16 E7 and clinicopathologic features were estimated, along with the relationship between VDAC1 and HPV16 E7 expression in cervical cancer specimens. **A.** Representative figures of VDAC1 and HPV16 E7 expression are displayed in a row, with the figures on the left representing low expression, figures in the middle representing light positive expression, and figures on the right representing positive expression (magnification 400X). **B.** The relative expression of HPV16 E7 in cervical cancer tissues with different invasive depths. **C.** The relative expression of HPV16 E7 in cervical cancer tissues according to FIGO staging. **D.** The relative expression of HPV16 E7 in cervical cancer tissues with or without metastasis. **E.** The relative expression of HPV16 E7 in cervical cancer tissues according to differentiation status: G1, well-differentiated; G2, moderately differentiated; G3, poorly differentiated. **F.** The relative expression of VDAC1 in cervical cancer tissues grouped according to HPV16 E7 expression. *, P< 0.05. **G.** The linear relationship between VDAC1 and HPV16 E7 expression. P > 0.05. The data are presented as the mean ± SD with scatter plots representing the individual detailed protein levels.

HPVs cause over 99% of cervical cancer cases, and HPV E6 and E7 may contribute to cancer progression. To further investigate the relationship between VDAC1 and HPV16 E7 in cervical cancer, we first detected the basal expression of HPV16 E7 in cervical cancer tissues. The expression of HPV16 E7 in these tissues was not related to invasion (Figure 3B), staging (Figure 3C) or lymph node metastasis (Figure 3D), but was significantly lower in well-differentiated cases (G3) than in poorly differentiated ones (G1 and G2) (Figure 3E).

When we analyzed the relationship between VDAC1 and HPV16 E7 expression (Figure 3G), we did not find a significant correlation (p > 0.05). The relationships of VDAC2 / VDAC3 with HPV16 E7 were also determined and are shown in Supplementary Figure 3. When the tissue samples were divided into groups based on HPV16 E7 expression, the expression of VDAC2 differed significantly between the low group and medium group (P = 0.0429), as well as between the medium group and high groups (P = 0.0453). However, VDAC3 expression was not related to HPV16 E7 expression (P = 0.0666).

### VDAC1 expression was positively related to HPV16 E7 expression in normal tissues and CIN tissues

**Figure 4:**
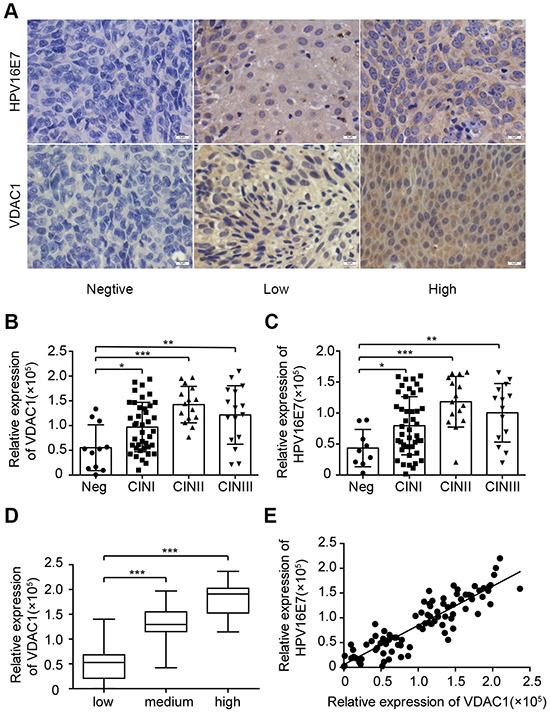
VDAC1 / HPV16 E7 levels correlated with clinicopathologic features and each other in CIN The levels of VDAC1 and HPV16 E7 in CIN specimens were measured by IHC. **A.** Representative figures of VDAC1 and HPV16 E7 expression are displayed in a row, with the figures on the left representing negative expression, figures in the middle representing light positive expression, and figures on the right representing positive expression (magnification 400X). **B.** The relative expression of VDAC1 in each group according to the severity of CIN progression. *, P < 0.05, ***, P < 0.001. **C.** The relative expression of HPV16 E7 in each group according to the severity of CIN progression. **D.** The expression of VDAC1 in each group, classified by the expression of HPV16 E7 in CIN tissues. **E.** The linear relationship between VDAC1 and HPV16 E7 expression in CIN. The data are presented as the mean ± SD with scatter plots representing the individual detailed protein levels.

Because HPV16 E7 promotes the progression of cervical cancer, we assessed the expression of VDAC1 and HPV16 E7 in precancerous (CIN) and normal tissues using IHC staining. As CIN progressed, the protein expression of VDAC1 and HPV16 E7 increased gradually, especially in CINI and CINII (Figure 4B-4C). Interestingly, the expression of VDAC1 correlated significantly with the expression of HPV16 E7 in CIN (Pearson's r = 0.86, P < 0.0001) (Figure 4D-E). Thus, the expression of VDAC1 may be induced by HPV16 E7 during the progression of CIN.

### In HPV16 E7-positive cell lines, VDAC1 expression correlated positively with HPV16 E7 expression

**Figure 5:**
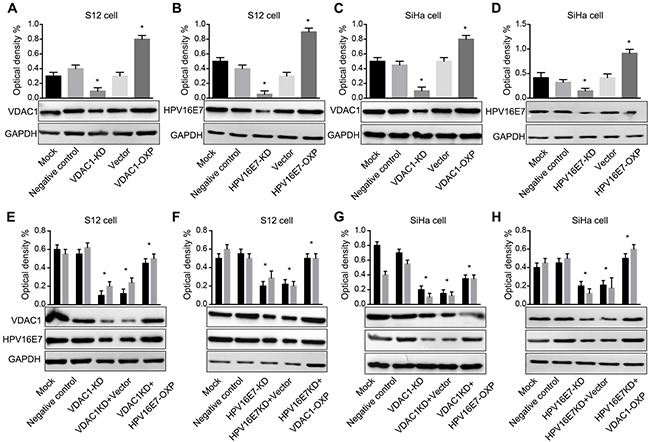
VDAC1 and HPV16 E7 protein levels were impacted in S12 and SiHa cell lines transfected with the corresponding CRISPR-VDAC1 or CRISPR-HPV16 E7 and overexpression plasmids Western blots are shown for **A.** VDAC1 protein expression in S12 cells transfected with CRISPR-VDAC1 and an ectopic VDAC1 plasmid. **B.** HPV16 E7 expression in S12 cells transfected with CRISPR-HPV16 E7 and an ectopic HPV16 E7 plasmid. **C.** VDAC1 expression in SiHa cells transfected with CRISPR-VDAC1 and an ectopic VDAC1 plasmid and **D.** HPV16 E7 expression in SiHa cells transfected with CRISPR-HPV16 E7 and an ectopic HPV16 E7 plasmid. Western blots are shown for **E.** VDAC1 and HPV16 E7 expression in S12 cells co-transfected with CRISPR-VDAC1 and an ectopic HPV16 E7 plasmid. **F.** VDAC1 and HPV16 E7 expression in S12 cells co-transfected with CRISPR-HPV16 E7 and an ectopic VDAC1 plasmid. **G.** VDAC1 and HPV16 E7 expression in SiHa cells co-transfected with CRISPR-VDAC1 and an ectopic HPV16 E7 plasmid and **H.** VDAC1 and HPV16 E7 expression in SiHa cells co-transfected with CRISPR-HPV 16 E7 and an ectopic VDAC1 plasmid. For all panels, quantitative measurements are shown above the Western blots. The expression of GAPDH was used for normalization. All data are presented as the mean ± SD of three independent experiments, * P < 0.05.

To test our hypothesis, we used specific Clustered Regularly Interspaced Short Palindromic Repeat sequences (CRISPR) to knockdown the protein expression of HPV16 E7 (CRISPR-HPV16 E7) or VDAC1 (CRISPR-VDAC1) in HPV16-positive cell lines. Conversely, VDAC1 and HPV16 E7 were overexpression by corresponding overexpression plasmids (Figure 5A-5D). Following knockdown or overexpression, the levels of VDAC1 and HPV16 E7 were assessed by Western blot. In these assays, CRISPR-VDAC1 markably reduced the protein expression of VDAC1 in S12 and SiHa cell lines, while VDAC1 overexpression plasmid increased the protein expression of VDAC1. Likewise, CRISPR-HPV16 E7 reduced HPV16 E7 expression, and the ectopic plasmid of HPV16 E7 increased its expression in S12 cell and SiHa cell lines.

Based on the results described above, we overexpressed HPV16 E7 when the expression of VDAC1 was blocked by CRISPR-VDAC1 in S12 and SiHa cell lines, and vice versa. As shown in Figure 5E-5H, the expression of VDAC1 was partly rescued when HPV16 E7 was overexpressed, and the expression of HPV16 E7 was partly rescued when VDAC1 was overexpression. As VDAC1 expression was induced by HPV16 E7 expression, it may be that, like HPV16 E7, VDAC1 acts as an oncoprotein in cervical cancer.

**Figure 6:**
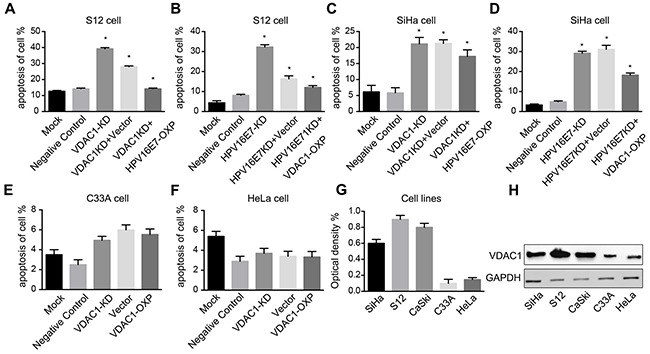
Reduced VDAC1 / HPV16 E7 expression induced apoptosis in cervical cancer cells transfected with CRISPR-VDAC1 or CRISPR-HPV16 E7, while overexpression of the HPV16 E7 / VDAC1 partly reversed these effects S12 and SiHa cell lines were transfected for 48 hours with the indicated CRISPR-VDAC1 / CRISPR-HPV16 E7 and corresponding overexpression plasmids. **A.** Apoptosis of the S12 cell line after co-transfection with CRISPR-VDAC1 and the HPV16 E7 plasmid. **B.** Apoptosis of the S12 cell line after co-transfection with CRISPR- HPV16 E7 and the VDAC1 plasmid. **C.** Apoptosis of the SiHa cell line after co-transfection with CRISPR-VDAC1 and the HPV16 E7 plasmid. **D.** Apoptosis of the SiHa cell line after co-transfection with CRISPR- HPV16 E7 and the VDAC1 plasmid. **E.** Apoptosis of the C33A cell line after co-transfection with CRISPR-VDAC1 and the VDAC1 plasmid. **F.** Apoptosis of the HeLa cell line after co-transfection with CRISPR-VDAC1 and the VDAC1 plasmid. **G.** The quantitative measurement of VDAC1 expression in cell lines is shown. (H) VDAC1 expression in cell lines were detected by Western blotting. The expression of GAPDH was used for normalization. All data are presented as the mean ± SD of three independent experiments, * P < 0.05.

Our previous data indicated that knockdown of HPV16 E7 could induce apoptosis in HPV16 E7-positive cells [22, 23]. To determine whether downregulation of VDAC1 would induce apoptosis in cervical cell lines, we used CRISPR-VDAC1 to block the expression of endogenous VDAC1 in human cervical cancer S12 and SiHa cells. The rate of apoptosis increased (39.4%) in S12 cells incubated with CRISPR-VDAC1, while overexpression of HPV16 E7 significantly reduced the rate of apoptosis to 14.7%. Similarly, when HPV16 E7 was knocked down with CRISPR-HPV16 E7, the apoptosis rate of S12 cell was 32.9%, while overexpression of VDAC1 reduced the apoptosis rate to 11.2%. Similar results were obtained in SiHa cells after incubation with the corresponding CRISPR-HPV16 E7 or CRISPR-VDAC1 plasmids. The detailed data were showed in Figure 6A-6D. These data suggested that knockdown of VDAC1 could increase the rate of apoptosis in cervical cancer cells, while the cell could be rescued by the overexpression of HPV16 E7.

### VDAC1 expression was not related to the fate of HPV16-negative cell lines

Considering that VDAC1 expression protected cervical cells against apoptosis, we wondered whether knocking down the expression of VDAC1 would impact the fate of HPV16-negative cells, such as HeLa and C33A. We first evaluated VDAC1 expression in cervical cancer cells (SiHa, S12, CaSki, C33A and HeLa). As shown in Figure 6G-6H, the protein level of VDAC1 was lower in HPV16-negative cells than in HPV16-positive cells. We then transfected the HPV16-negative C33A and HeLa cells with CRISPR-VDAC1 and the VDAC1 overexpression plasmids. As shown in Figure 6E-6F, the apoptosis rates of the two cell lines did not differ significantly from that of the control group. These results indicated that the expression of VDAC1 is not as important in HPV16-negative cells as it is in HPV16-positive cells.

## DISCUSSION

Cervical cancer is the second most frequent carcinoma among women [24]. Persistent HPV infection and integration are the major pathogenic factors of cervical cancer [25–27], However, HPV infection is necessary but not sufficient to induce cancer. Oncoprotein E7 contribute to the primary transforming activity and cooperate in the progression of HPV-induced cancers [28]. It was reported that HPV16 E7 attenuated DNA damage checkpoint control in infected cells [29], suggesting that E7 may promote oxidative damage.

VDAC1, the main channel of the mitochondrial outer membrane, anchors different pro- and anti-apoptotic proteins [10]. As it functions at the crossroads of metabolic and survival pathways, VDAC1 seemed to be a promising mitochondrial target for cancer therapy. However, we did not find any relationship between the expression of VDAC1 and HPV16 E7 in cervical cancer tissues (Figure 3), consistent with the arguments of Guo-Qing, et al. [30]. On the other hand, the expression of VDAC1 was confirmed to correlate significantly with that of HPV16 E7 in the CIN tissues (Figure 4). These data indicated that VDAC1 may work in coordination with HPV16 E7, which promotes the progression of precancerous cells.

VDAC1 is an important agent in cell homeostasis and ROS [31]. In the present research, we found that the expression of VDAC1 only correlated with that of E7 in precancerous tissues, which indicated that E7 may promote ROS production in the early stages of HPV infection. De Marco et al. reported that protein carbonyls were significantly more abundant in dysplastic tissues than in normal tissues, while the protein oxidation levels were surprisingly similar in cancerous and normal tissues. These changes were paralleled by the extent of oxidative DNA damage, as 8-OH-dG levels were markedly greater in dysplastic tissues than in either cervical cancer or normal tissues [32]. These findings strongly suggested that a precancerous state in the cervix is highly vulnerable to oxidative damage, which was consistent with our previous conclusion.

We found that all three isoforms of VDAC were significantly oxidized in HPV-positive cervical cancer cells. VDAC1 expression correlated significantly with the invasion of clinical cervical cancer (Figure 2A). Knockdown of VDAC1 expression was previously shown to reduce the mitochondrial membrane potential, adenosine triphosphate levels and migration of cell lines [33]. The expression of the other two members, VDAC2 and VDAC3, correlated with the metastasis of cervical cancer in the present study. Although all three isoforms are expressed ubiquitously in all tissues, the relative amount of VDAC1 is the greatest among the isoforms.

We first used an OxICAT approach to detect the global redox status of proteins in HPV-related cervical cancers. Except for the candidate proteins VDAC1, VDAC2 and VDAC3, the other selected proteins, (RAB30 [34], PON2 [35], SUN1 [36], GNPNAT1 [37], KNTC1 [38], EML4 [39], AP3M1 [40], and PGRMC2 [41]), were all associated with cellular energetics and cell survival, but we did not find any differences in their oxidation between HPV-positive and HPV-negative cell lines. This may have been because we analyzed cervical cancer cells rather than CIN cell lines. Indeed, the expression of VDAC1 only correlated with HPV16 E7 in precancerous tissues, not in cancerous tissues. The oxidation of these energy-associated proteins in precancerous tissues should be studied further.

In summary, the present study indicated that VDAC1 may interact with HPV16 E7 to promote the malignant progression of HPV-related disease, especially in the precancerous stage, and that the expression of VDAC1 correlated with the invasion of cervical cancer. These data suggested that suppression of VDAC1 could be a potential strategy by which to block the progression of HPV-related cervical disease, especially in CIN.

## MATERIALS AND METHODS

### Tissue samples and cervical cancer cell lines

With approval from the institutional review board, we obtained cervical cancer patients’ cancer tissue samples via surgery from the Department of Obstetrics and Gynecology of Tongji Hospital, which is affiliated with Huazhong University of Science and Technology, between 2012 and 2014. Two different pathology experts determined the pathological diagnoses of all tissue samples. Ultimately, we obtained 57 cervical cancer tissue specimens (all of which were assessed for HPV16 positivity with a PCR test). Written informed consent was obtained from all patients enrolled in the study. Cervical cancer cell lines (SiHa, C33A, CaSki and HeLa) were purchased from the American Type Culture Collection. All cell lines were cultured at 37°C in a humidified atmosphere containing 5% CO_2_. The S12 cell line is an immortalized human cervical keratinocyte cell line that contains integrated HPV16 genomes, which was a generous gift from Professor Ken Raj (Health Protection Agency). The acquisition of the cell line was permitted by the original owner Professor Margaret Stanley [42, 43]. S12 cells were cultured in a 1:3 mixture of Dulbecco's Modified Eagle Medium (DMEM) and Ham F-12 medium supplemented with 5% fetal bovine serum (FBS), 8.4 ng/mL cholera toxin, 5 μg/mL insulin, 24.3 μg/mL adenine, 0.5 μg/mL hydrocortisone, and 10 ng/mL epidermal growth factor (EGF).

### Tissue micro-array (TMA) block construction

All cervical cancer tissue specimens were constructed into TMA blocks as follows: hematoxylin and eosin-stained slides from all the formalin-fixed, paraffin-embedded tissue blocks were examined by a pathologist, who located representative areas of cervical cancer. From each block, one tissue biopsy (1-mm diameter) was placed on the recipient TMA block, 4-μm-thick slides were obtained from the TMA block, and the TMA slides were assessed again for the original lesion and used for the subsequent experiment.

### OxICAT

The global oxidized status of proteins was detected by OxICAT as previously described [44]. Briefly, total proteins in the cells were denatured, and the proteins were incubated with light ICAT (^12^C-ICAT) for irreversible labeling of all the reduced cysteines. Next, the strong thiol reductant Tris(2-carboxyethyl)phosphine were used to reduce all the reversible oxidative thiol modifications of the samples, and these newly accessible cysteines were labeled with heavy ICAT (^13^C-ICAT). All the ICAT-labeled peptides were digested, affinity-purified and quantified with mass spectrometry.

### BIAM

Cells were lysed for 15 min. on ice in biotin labelinglysis buffer (BLLB: 50 mM Tris-HCl pH 7.0, 5 mM ethylenediaminetetraacetic acid (EDTA), 120 mM NaCl, 0.5% Igepal-630) containing protease inhibitors and 100 mM maleimide (Sigma, 129585, China). Insoluble material was then removed by centrifugation at 20,000xg for 10 min. at 4°C, the cleared supernatant was transferred to a fresh Eppendorf tube and the protein concentration was determined by the Bradford assay. The protein concentration was adjusted to 1 μg/μl with BLLB, sodium dodecyl sulphate (SDS) was added from a 10% stock to a final concentration of 1% and the cell lysates were rotated at room temperature for two hours. For the removal of unreacted maleimide, proteins were subsequently precipitated by the addition of five volumes of acetone pre-equilibrated at −20°C and then were incubated for 20 min. at −20°C. The preparations were centrifuged at 20,000x*g* for 10 min. at 4°C, the supernatants removed and discarded and the precipitated protein pellet was air-dried. The pellet was then resuspended in 200 μl BLLB containing 1% SDS, 10 mM dithiothreitol (DTT) and 0.1 mM biotin-maleimide (Sigma-B1267, stock dissolved in dimethylformamide) so that to the remaining, previously oxidized, sulfhydryl groups would be reduced and allowed to react with biotin-maleimide. Proteins were again precipitated with five volumes of methanol (−20°C) as above, and the dried pellet was resuspended in 500 μl of BLLB, and rotated at 4°C for two hours with 10 μl of a 50% slurry of streptavidin-sepharose beads (GE Healthcare-17511301). The beads were then washed four times with BLLB and resuspended in SDS-polyacrylamide gel electrophoresis (PAGE) loading buffer for SDS-PAGE analysis and Western blotting.

### IHC

IHC reactions were performed on TMA slides as described elsewhere. The TMA slides were incubated overnight in a humidified chamber at 4°C with primary antibodies: rabbit anti- VDAC1, anti-VDAC2, and anti-VDAC3 (1:300, Proteintech, USA) and rabbit anti-HPV16 E7 (1:200, Biorbyt). Antibody detection was performed with 3,3′-diaminobenzidine (DAB). CellSens Dimension (version 1.8.1, Olympus) were used to photograph the images and the staining intensity was measured with ImagePro Plus (version 6.0, Media Cybernetics).

### CRISPR technology to knock down the expression of VDAC1 and HPV16 E7 in cervical cancer cell lines

The CRISPR transfection system and all alignments were constructed by Dr. Hu in our lab (the sequences are displayed below). The HPV16 E7 expression plasmid was a gift from Harald zur Hausen (German Cancer Research Center, University of Heidelberg, Heidelberg, Germany) and the specific overexpression plasmid targeting VDAC1 was constructed by GeneCreate company (Wuhan). The X-tremeGENE HP reagent (Roche) were used according to the manufacturer's instructions to transfect human cervical cancer cell lines (SiHa, S12, C33A and HeLa) with gRAN-VDAC1/ gRAN-HPV16 E7 or green fluorescent protein (GFP) plasmids. The ratio of the reagent to DNA for each cell line was optimized in preliminary experiments. The experiments were performed in triplicate and repeated twice.

*gRNA-VADC1 binding sequence*: CAAGATCGGC ATACGTGGG

*gRNA-HPV16 E7 binding sequence*: GCTGGACA AGCAGAACCGGA

### Western blotting analysis

Cells were lysed in a buffer containing 50 mM Tris (pH 7.4), 150 mM NaCl, 1% Triton X-100, 1% sodium deoxycholate, 0.1% SDS, and a protease inhibitor cocktail (Beyotime). The primary antibodies used for Western blotting analysis included rabbit anti-HPV16 E7 (1:200, Biorbyt), rabbit anti-VDAC1 (1:300, Proteintech, USA), and rabbit anti-GAPDH (1:3000, Proteintech, USA). Anti-GAPDH was used as an internal standard for all samples. The signals were detected with Lab image (Beyotime, China).

### Apoptosis assay by Annexin V/PI staining

An Annexin V-FITC Apoptosis Kit I (BD, San Jose, CA, USA) was used to detect apoptosis. Samples were acquired on a FACS can flow cytometer (FACS Calibur, Becton Dickinson) and analyzed with CELL Quest software. All procedures were performed according to the manufacturer's instructions.

### Statistical analysis

Analyses were performed with Image-Pro Plus and GraphPad Prism 5. The data are presented as the mean ± SD. A two-tailed Student's t test was used to determine the significance of differences between the treated and control groups. A P value of less than 0.05 indicated a significant difference between two groups.

## SUPPLEMENTARY FIGURES AND TABLES






