# Supplementary material for: Proteomics-based identification of VDAC1 as a tumor promoter in cervical carcinoma
Source: Oncotarget. 2016 Jul 13;7(32):52317–28. doi: 10.18632/oncotarget.10562 (PMC5239554; doi:10.18632/oncotarget.10562)
Supplement: Supplementary file 1 [file oncotarget-07-52317-s001.pdf]

Proteomics-based identification of VDAC1 as a tumor promoter in cervical carcinoma

SUPPLEMENTARY FIGURES AND TABLES

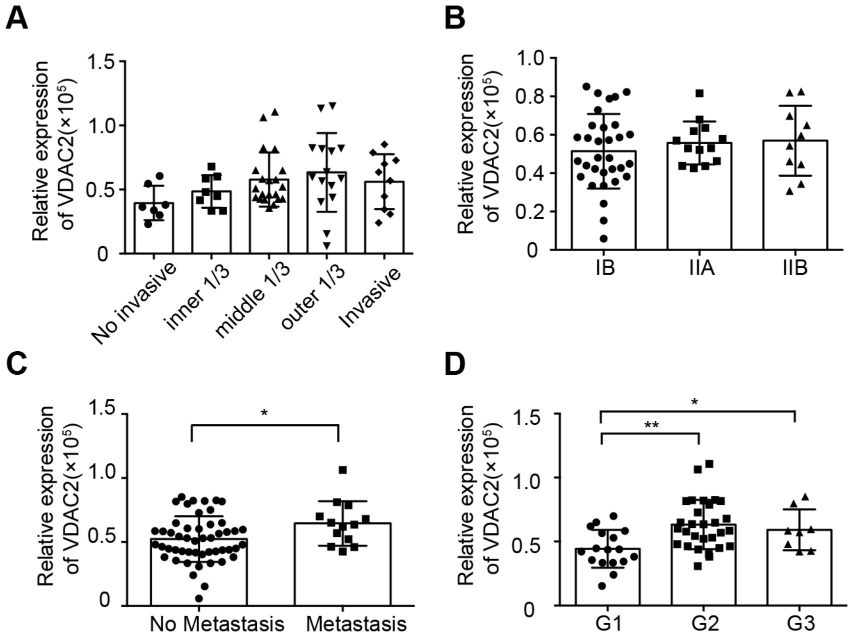

Supplementary Figure 1: VDAC2 expression correlated with the metastasis and differentiation status in cervical cancer. The expression of VDAC2 in cervical cancer specimens was detected by IHC staining. And the relationships between the levels of VDAC2 and clinicopathologic features were estimated. **A.** The relative expression of VDAC2 in cervical cancer tissues with different invasive depths. **B.** The relative expression of VDAC2 in cervical cancer tissues according to FIGO staging. **C.** The relative expression of VDAC2 in cervical cancer tissues with or without metastasis. *, $P < 0.05$. **D.** The relative expression of VDAC2 in cervical cancer tissues according to differentiation status: G1, well-differentiated; G2, moderately differentiated; G3, poorly differentiated. The data are presented as the mean ± SD with scatter plots representing the individual detailed protein levels.

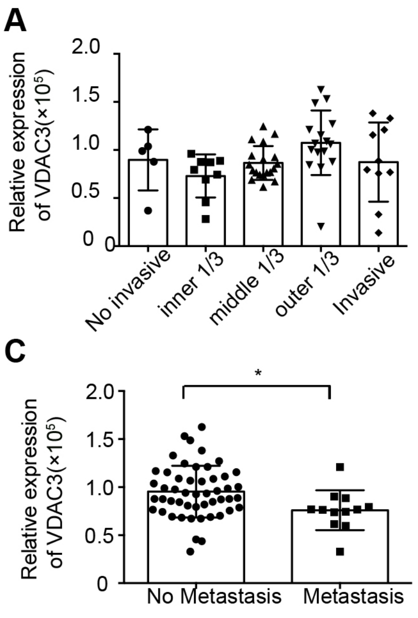

Supplementary Figure 2: VDAC3 expression correlated with the metastasis and differentiation status in cervical cancer. The expressions of VDAC3 in cervical cancer specimens was detected by IHC staining. And the relationships between the levels of VDAC3 and clinicopathologic features were estimated. **A.** The relative expression of VDAC3 in cervical cancer tissues with different invasive depths. **B.** The relative expression of VDAC3 in cervical cancer tissues according to FIGO staging. **C.** The relative expression of VDAC3 in cervical cancer tissues with or without metastasis. *, $P < 0.05$. **D.** The relative expression of VDAC3 in cervical cancer tissues according to differentiation status: G1, well-differentiated; G2, moderately differentiated; G3, poorly differentiated. The data are presented as the mean ± SD with scatter plots representing the individual detailed protein levels.

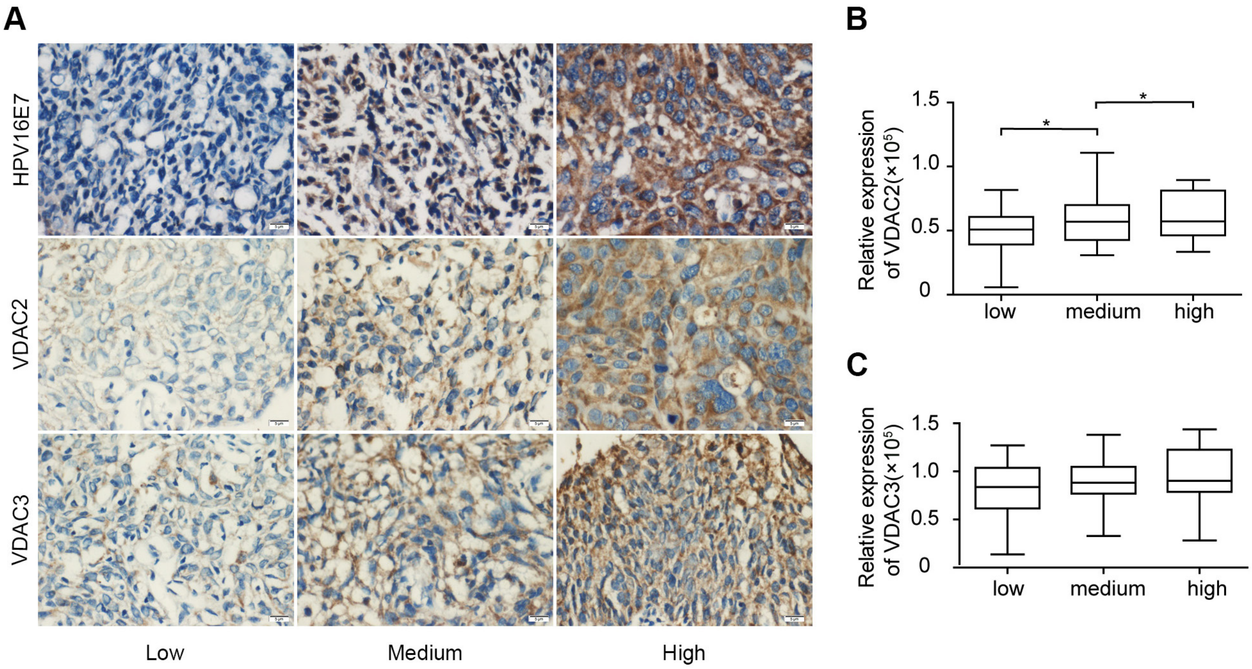

Supplementary Figure 3: VDAC2 expression correlated with HPV16 E7 expression in cervical cancer. The relationships between the expression of VDAC2/VDAC3 and HPV16 E7 were estimated. **A.** Representative figures of VDAC2/VDAC3 and HPV16 E7 expression were displayed in a row, with the figures on the left representing negative or low expression,figures in the middle representing light positive expression, and figures on the right representing positive expression (magnification 400X). **B.** The relative expression of VDAC2 in cervical cancer tissues grouped according to HPV16 E7 expression. *, $P < 0.05$. **C.** The relative expression of VDAC3 in cervical cancer tissues grouped according to HPV16 E7 expression. *, $P < 0.05$.

Supplementary Table S1: The detailed clinicopathological parameters of the normal tissues and CIN tissues used in this assay.

See Supplementary File 1

Supplementary Table S2: The detailed clinicopathological parameters of the cervical cancer tissues used in this assay.

See Supplementary File 2
